# Supplementary material for: Calculating worldwide needs for morphine for pain in advanced cancer and proportions feasibly met by country estimates of requirements and consumption. Retrospective, time-series analysis (1997–2017)
Source: PLOS Glob Public Health. 2022 Jul 8;2(7):e0000533. doi: 10.1371/journal.pgph.0000533 (PMC10021698; doi:10.1371/journal.pgph.0000533)
Supplement: S1 Fig — (DOCX) [file pgph.0000533.s001.docx]

*S1 Fig: Proportion of included countries reporting consumption which could feasibly meet > or ≤ 100% of calculated need for morphine of people with advanced cancer, 1997-2017*
