# Supplementary material for: Calculating worldwide needs for morphine for pain in advanced cancer and proportions feasibly met by country estimates of requirements and consumption. Retrospective, time-series analysis (1997–2017)
Source: PLOS Glob Public Health. 2022 Jul 8;2(7):e0000533. doi: 10.1371/journal.pgph.0000533 (PMC10021698; doi:10.1371/journal.pgph.0000533)
Supplement: Attachment — Submitted filename: Response to reviewers.docx [file pgph.0000533.s004.docx]

| Editor |  |  |
| --- | --- | --- |
| 1 | This study adds immense value to a rather underrepresented aspect of global public health in oncology. I congratulate the authors for their commendable work. Look forward to your submission in response to the revisions suggested. | Thank you. |
| 2 | Guidelines for resubmitting your figure files are available below the reviewer comments at the end of this letter. | Thank you, we have done so. |
| 3 | 1. Your co-authors, Miriam J Johnson (miriam.johnson@hyms.ac.uk), Christina Ramsenthaler (christina.ramsenthaler@hyms.ac.uk), and David C Currow (david.currow@uts.edu.au), have not confirmed authorship of the manuscript. We have resent them the authorship confirmation email; however please check that the above email address for them is correct and follow up personally to ensure they confirm. Please note that we cannot pass your manuscript to Production until we have received confirmations from all co-authors.  Just in case your co-authors are having difficulty confirming their authorship, you may advise them to send us an email at globalpubhealth@plos.org and we will confirm their authorship on the authors' behalf. | Thank you – all authors have been reminded to confirm authorship using the link provided to them. Professor David Currow has requested that his institution and e-mail address and I will e-mail [globalpubhealth@plos.org.uk](mailto:globalpubhealth@plos.org.uk) on his behalf |
| 4 | 2. Please provide a detailed Financial Disclosure statement. This is published with the article, therefore should be completed in full sentences and contain the exact wording you wish to be published.  i) Please include all sources of funding (financial or material support) for your study. List the grants (with grant number) or organizations (with url) that supported your study, including funding received from your institution.  ii). State the initials, alongside each funding source, of each author to receive each grant.  iii). State what role the funders took in the study. If the funders had no role in your study, please state: “The funders had no role in study design, data collection and analysis, decision to publish, or preparation of the manuscript.”  iv). If any authors received a salary from any of your funders, please state which authors and which funders.  If you did not receive any funding for this study, please simply state: “The authors received no specific funding for this work.” | Thank you. We have updated the section to read:  “The authors received no specific funding for this work.” |
| 5 | 3. Please update your Competing Interests statement. If you have no competing interests to declare, please state: “The authors have declared that no competing interests exist.” | Thank you. We have updated the section to read: “The authors have declared that no competing interests exist.” |
| 6 | 4. Please note that your Data Availability Statement is currently missing the repository name and/or the DOI/accession number of each dataset OR a direct link to access each database. If your manuscript is accepted for publication, you will be asked to provide these details on a very short timeline. We therefore suggest that you provide this information now, though we will not hold up the peer review process if you are unable. | Thank you. We have updated the statement as follows:  “All data used are available in the public domain using references provided in the manuscript.” |
| 7 | 5. We do not publish any copyright or trademark symbols that usually accompany proprietary names, eg (R), (C), or TM (e.g. next to drug or reagent names). Therefore please remove all instances of trademark/copyright symbols throughout the text, including IBM® SPSS® on page 9. | Done |
| Reviewer 1 |  |  |
| 8 | This is an important work and highlights a neglected issue in LMIC context. |  |
| 9 | Overall paper is hard to read and follow as the terms need, requirement, theoretical availability and consumption are very hard to understand. Authors need to give clear definitions and use consistent language to make it understandable. It will useful to use only 2-3 key indicators to explain trends. | We have clarified and standardised use of our key terms (point 12). We have also conducted a full copy edit for readability and removed an indicator (statistical relationship between income group and proportions of treatable patients using estimates of requirements) |
| 10 | The paper uses very important concepts from supply chain management which may not be well understood by medical / public health audience. Introduce the audience to key supply chain management concepts relevant to the paper such as forecasting, availability and consumption. | In the Introduction, we introduce the question of whether estimates of requirements contextualise consumption. We answer this question in the Results section and note that ““obtaining accurate information about the legitimate requirements for [controlled] substances is a prerequisite to ensuring their availability [p.iv, 2012].””  As this issue is about the legal and regulatory frameworks rather than about manufacture and distribution, we believe further detail regarding supply chain/production etc. is out of scope here. However, we have added further detail regarding how consumption data are gathered, clarifying the link between international and national regulations in terms of consumption as follows:  “Methods with which individual countries calculate *estimates of requirements* are unreported. However, at the service-level, reporting of consumption to national regulators of controlled substances is a prerequisite to obtaining and maintaining a license to prescribe opioids. A controlled substance is regarded as “consumed” when it has been supplied to any person or enterprise for retail distribution, medical use or scientific research; and “consumption” is construed accordingly.^4^” |
| 11 | Operational definitions in methods: Estimated need, estimated requirement, theoretical availability and consumption should be clearly defined in the methods section. Ensure use of consistent terminology in methods, results and tables for clear understanding. | The authors recognise the need to be clear about a number of key terms used throughout the manuscript and also dropped ‘theoretically treatable’ and replaced this with ‘feasibly treatable’ to aid clarity. We have therefore added a section defining key terms as follows:  Key terms  Our study uses a number of related but distinct key terms throughout the manuscript. Key terms are presented and defined in Box 2:  *Estimates system* – refers to the INCB’s method of implementation of the Single Convention on Narcotics, which requires countries to submit Estimates of Requirements for controlled substances annually.  *Estimates of requirements* – refers to estimated or calculated quantities of controlled substances individual countries submit to the INCB annually to provide medical treatment through **existing** health-care infrastructure.*  *Consumption* – refers to quantities of controlled substances reported annually to the INCB as having been ‘consumed.’ Controlled substances are considered to have been ‘consumed’ where it has been supplied to any person or enterprise for retail distribution, medical use or scientific research.  *Calculated need* – refers to estimates of country-level need for morphine for people with advanced cancer at INCB-recommended dosage and duration used in this study (80% of people who die from cancer, 67.5mg per day for the last 90 days of life)  *Total/feasibly treatable* – refers to total or proportion of *calculated need* feasibly met using *Estimates of requirements* or *Consumption* assuming that all morphine is administered to people who die from cancer at INCB-recommended dosage and duration.  *Estimates of requirements are not equivalent to country estimates of clinical needs. The INCB notes that “in an ideal system, the requirements for controlled substances would equal the needs [INCB, p6].” ^4^  Additionally, we standardised use of key terms throughout the manuscript and have italicised all use of key terms to aid clarity for the reader. |
| 12 | • Box 1 is very useful. Connect the methods given in box to indicators. Estimated need, estimated requirement, theoretical availability –mention which is based on consumption and which is based on morbidity method. | Methods of estimating requirements for morphine recommended by the INCB are presented in Box 1. We have made no changes to the Box, but have made clear in the manuscript that we use only an adapted morbidity-based method and have made a change to our aims as follows:  We aimed to calculate needs for morphine for people who die from cancer using an adapted morbidity-based method [Box 1] and identify changes in proportions of calculated need which could theoretically be met by countries’ submitted *estimates of requirements* using INCB-recommended methods of calculation between 1997 and 2017. |
|  | Table 1 : Estimated requirement for year 2002 for low and low middle income countries is very high compared to subsequent years. Explain in results and discussion. | This is explained in the table’s footnote “** A large spike in estimated requirement in this year is accounted for by a large increase in a single country’s estimate of requirement for morphine in this year.” – However, the ** was omitted from the table to indicate this spike – this omission has now been corrected. We have also added the following text explaining the spike in the estimate of requirement.  “For all country groups, fluctuations are present in population, calculated needs for morphine and estimates of requirement between time-points, notably in low income and upper-middle income countries. Fluctuations are accounted for both by changes to individual countries’ estimate of requirement and differences in countries included within each income group, either due to some countries not submitting estimates of requirements for individual years, or movement between income group of countries.”  We have also added the following paragraph to our study limitations:  “Additionally, our use of a repeated time-series approach means that total countries included at each time point differ by year. This explains some of the fluctuation in our outcome variables presented in Table 1. For example, there is notable fluctuation in countries’ population, calculated need and estimate of requirements in low income countries, between 1997, 2002 and 2007. This is accounted for by an increase of six countries in the sample from 1997 to 2002 and a significant increase in India’s estimates of requirement between the dates, from 400kg in 1997, to 10,000kg in 2002. India was included as a lower-middle income country by 2002, where after total estimate of requirement in low income countries dropped to similar levels seen in 1997 and remained consistent.” |
| 13 | Table 1 : In the upper middle income countries, estimated requirement follows a fluctuating patterns. Explain in results and discussion. | See response to Item #12 above. |
| 14 | Line 201- 205 – Need vs requirement not clear | We have clarified and italicised the language used here in line with our now defined key terms:  “Fig 3 shows the proportion of total countries submitting *estimates of requirements* which could technically meet and exceed *calculated needs* for morphine of people with advanced cancer, increased: 1997 (28/176, 16%), 2002 (43/181, 24%), 2007 (42/186, 23%), 53/186, 28%) and 2017 (57/187, 30%).” |
| 15 | Table 2 interpretation – line 231 -234 is not clear . Is this table adding to the overall conclusions? If not, it can be removed. | We have deleted this short section to make space for an additional table (see below). |
| 16 | Consumption of morphine Lines 239 -249 – This is an important para and should be supported by a table. | See response #17 below |
| 17 | Add a table which should compare consumption versus what countries had projected as the need and reported overall and if possible by country groups. | We have added an additional table on p14/15 which includes the comparison between percentage of calculated need feasibly met by estimates of requirements and consumption – including by income group. |
| 18 | Figure 4 and 5 – very hard to follow. Consider simplifying or explaining in the results | We have added the following additional text to help explain figure 4.  “The proportion of countries which submitted *estimates of requirements* sufficient to meet >100% of *calculated needs* increased from 16% in 1997 to 30% in 2017. This indicates that an increasing proportion of world countries are submitting *estimates of requirements* for morphine which exceeds quantities necessary to treat people who die from advanced cancer at INCB-recommended dosage and duration, with additional morphine then available for other approved clinical indications such as post-operative and trauma analgesia.”  We have also modified the explanation of Figure 5 to better explain the figure:  “The ~~proportion~~ number of countries within income groups changes within our sample. However, the proportion of countries within each income group submitting *estimates of requirements* which could theoretically meet *calculated need*s for morphine for cancer-related pain relief, with additional capacity to treat others requiring morphine, changes little between 1997 and 2017 in any income group.”  We have additionally altered the name of Figure 5 to aid clarity:  “Fig 5: Proportion of countries within each income group submitting estimates of requirements able to meet ≤ or > 100% of calculated need by year and income group” |
| 19 | Lines 265 – 278 Discussion section explains results. Consider moving it to results | We do reiterate some results in this section but are satisfied that there is additional interpretation here and have left the section as discussion, |
| 20 | While revising your submission, please upload your figure files to the Preflight Analysis and Conversion Engine (PACE) digital diagnostic tool, https://pacev2.apexcovantage.com/. PACE helps ensure that figures meet PLOS requirements. To use PACE, you must first register as a user. Registration is free. Then, login and navigate to the UPLOAD tab, where you will find detailed instructions on how to use the tool. If you encounter any issues or have any questions when using PACE, please email PLOS at figures@plos.org. Please note that Supporting Information files do not need this step. | All files have been converted to TIF files using this tool and reuploaded. |
